# Status and Power Do Not Modulate Automatic Imitation of Intransitive Hand Movements

**DOI:** 10.1371/journal.pone.0151835

**Published:** 2016-04-20

**Authors:** Harry Farmer, Evan W. Carr, Marita Svartdal, Piotr Winkielman, Antonia F. de C. Hamilton

**Affiliations:** 1 Institute of Cognitive Neuroscience, University College London, 17 Queen Square, London, WC1N 3AR, United Kingdom; 2 Department of Psychology, University of California, San Diego, 9500 Gilman Drive, 0109 La Jolla, CA 92093-0109, United States of America; 3 Behavioural Science Group, Warwick Business School, The University of Warwick, Coventry, CV4 7AL, United Kingdom; 4 Department of Psychology, SWPS University of Social Sciences and Humanities, ul. Chodakowska 19/31, 03-815, Warsaw, Poland; University of Bologna, ITALY

## Abstract

The tendency to mimic the behaviour of others is affected by a variety of social factors, and it has been argued that such “mirroring” is often unconsciously deployed as a means of increasing affiliation during interpersonal interactions. However, the relationship between automatic motor imitation and status/power is currently unclear. This paper reports five experiments that investigated whether social status (Experiments 1, 2, and 3) or power (Experiments 4 and 5) had a moderating effect on automatic imitation (AI) in finger-movement tasks, using a series of different manipulations. Experiments 1 and 2 manipulated the social status of the observed person using an associative learning task. Experiment 3 manipulated social status via perceived competence at a simple computer game. Experiment 4 manipulated participants’ power (relative to the actors) in a card-choosing task. Finally, Experiment 5 primed participants using a writing task, to induce the sense of being powerful or powerless. No significant interactions were found between congruency and social status/power in any of the studies. Additionally, Bayesian hypothesis testing indicated that the null hypothesis should be favoured over the experimental hypothesis in all five studies. These findings are discussed in terms of their implications for AI tasks, social effects on mimicry, and the hypothesis of mimicry as a strategic mechanism to promote affiliation.

## Introduction

People’s tendency to spontaneously mimic others’ actions has been described as a form of “social glue” [1], which is used strategically to increase affiliation with others [2,3]. Increasing evidence also suggests that mimicry can be modulated by a variety of social factors, including prosociality, affiliation goals, eye contact, and self-other distinction [4–8].

With the current paper, we explore whether mimicry is also modulated by the *status* (i.e., objective prestige or advancement) or *power* (i.e., subjective feelings of control over resources) of their interaction partner. In what follows, we first detail and describe several different types of mimicry that have previously been investigated. Next, we briefly review the findings on the social modification of mimicry, starting with more general social cues and then focusing more specifically on status and power. Finally, we offer a short outline of the five experiments presented here, along with our general goals for their design.

### Types of mimicry

Before viewing the current state of the literature, it is useful to clarify exactly what we define and discuss as mimicry. We distinguish between three different behavioural phenomena, which have all been considered as forms of mimicry in the literature.

The first type is automatic imitation (AI), which can be defined as a kind of stimulus-response compatibility (SRC) effect where the action stimulus (either video or image) is paired with a congruent or an incongruent response. For example, a participant might be instructed to use their index finger to press a key if they see a “1” appear on the screen, and use their middle finger to press another key if they see a “2” appear on the screen. During the task trials, they would then observe an index finger being lowered on the screen while seeing either a “1” (congruent) or a “2” (incongruent) as their own movement cue. The difference in reaction time between congruent and incongruent trials is taken as a measure of AI [9–11].

A second type of mimicry is often referred to as behavioural mimicry (BM), which can be defined as the tendency of people to naturally copy others’ movements during social interactions [1,8]. BM is generally measured using naturalistic observational paradigms, where the key measure is the extent to which participants copy particular movements made by a confederate (e.g. face-touching or foot-tapping). A third type of mimicry is called facial mimicry (FM), which describes the tendency to mimic others’ facial expressions [12]. FM is most commonly measured using electromyography, in order to measure subtle activation of different facial muscles.

It is currently unclear whether these three different types of mimicry share the same underlying mechanism, or if each relies on distinct neurocognitive processes. This is a complex question, for many reasons. As an example, many argue for the role of FM in other related (yet distinct) processes like emotional recognition and contagion [8]. Nevertheless, the relationship between BM and AI has received some consideration, most notably by Heyes [10], who argues that the SRC effects from AI studies differ from other similar effects, like spatial compatibility or emulation [13]. More specifically, Heyes argues that AI and BM utilise similar cognitive processes but have different behavioural outcomes (e.g., an analogy might be the relationship between silent reading and reading aloud). This view has been strengthened by studies demonstrating SRC effects during rhythmic movements [14] and extended motor sequences [15], which are closer to the complex motor behaviours in BM, compared to the basic discrete motor actions in AI. Having said this, the AI and BM phenomena clearly occur at different time scales (500 ms vs. several seconds, respectively), and thus, are differentially open to strategic modification and complex input. Critically though, note that AI and BM are also similar in the sense that both are modulated by social cues, a phenomenon we review in the next section.

### Mimicry and social cues

Increasing evidence shows that prosocial cues, such as eye contact and liking, have a strong impact on mimicry. For example, Wang et al. [5] found greater AI when participants’ interaction partner engaged in direct eye contact, compared to when the partner averted their gaze. This effect was specific to eye contact and did not occur when flash-box cues were placed in the centre or periphery of the screen, suggesting that attention alone could not account for the effect. Other studies have found that participants show greater FM to friends than strangers [16] and greater FM and BM to likeable rather than unlikeable strangers [16–18]. Consistent with these results, Lakin and Chartrand [19] observed greater BM among participants who had explicitly been told to affiliate with others.

Modulation of mimicry can also be seen when social relations are manipulated using more indirect methods. One such method is sentence priming. After unscrambling prosocial sentences, participants show greater AI [6] but only if those sentences are self-related [4]. Another example method is social exclusion. Being socially excluded from an in-group leads individuals to increase BM [20], while witnessing ostracism can actually cause children to *over*-imitate, possibly in order to increase their affiliation with the actor [21]. Finally, other experiments have demonstrated that participants show greater FM [22] and BM [23] to those from a similar ethnic, political, or religious background as themselves.

Taken together, these results suggest that mimicry is influenced by social relations between the perceiver and target (action displayer). One particularly important element appears to be the need or intent to affiliate with the target. Several theorists [2,3] have argued that mimicry is unconsciously and strategically deployed to increase affiliation, especially in situations where such affiliation would be valuable. Affiliation is particularly valuable during interactions with in-group members and situations following social exclusion. Findings where mimicry increases in these circumstances provide some evidence for the possible utilization of “smart” mimicry behaviour, which can become more or less strategic, depending on the social context (for a review and discussion see [24]). As such, mimicry clearly relies on domain-general contextual modulation processes to some extent, but also appears to reflect a basic form of social regulation. Many predictions can be made for how hierarchical cues of status and power may be involved, which we move on to next.

### The modulation of mimicry by social status and power

Social status and power are critical social cues that fundamentally change the relation between the perceiver and the target [25] and could thus potentially modulate mimicry behaviour toward others. While closely related, it is important to distinguish between *status* and *power*. Magee and Galinsky [26] define social status as “the extent to which an individual or group is respected or admired by others,” and social power as “holding asymmetric control over valued resources.” While it is often the case that high-status individuals also hold large amounts of power, it is clear that status and power are not always linked (e.g., traffic wardens hold relatively high-power in the context of parking, but relatively low-status in terms of more general social prestige). Such rankings are a ubiquitous feature of social organisation across species [27] and have a substantial impact on human social interaction [28]. A rich literature in social cognition has documented a variety of ways in which the power and status of social targets modifies not only perceivers’ motor behaviour but also various forms of cognitive processing [29,30]. The idea that mimicry is strategically used to improve an individual’s social standing suggests that social status and power may also play a modulating role on the tendency to mimic.

Note, however, that any potential effects of social status, power, and hierarchy on AI could be driven by either basic perceptual/cognitive processes or higher-level interpretational processes (or both). In terms of basic processes, status and power could possibly influence AI via “input” processes, such as attention or cognitive load [10]. For instance, Fiske and Deprét [31] suggest that people direct more attention to those in high-status positions because they are in control of resources that could potentially alter people’s outcomes. Some studies lend support to this claim, given that people display greater favouritism towards high-status groups [32] and more deference towards high-status individuals [33–35]. Participants also more closely track the eye gaze of high-status individuals [36] and have a tendency towards greater recall of high-status faces [37]. Moreover, Dalton, Chartrand, and Finkel [38] showed that being mimicked by a high-status other or mimicking a low-status other led to a greater depletion of cognitive resources than the reverse, suggesting that participants found it less cognitively demanding when higher-status targets were imitated. Another basic process that might affect AI is inhibitory or “output” modulation [10]. Here, social factors could lead to an inhibition or facilitation of motor response to the observed motor action. The output modulation account fits with the result of a recent study by Hogeveen, Inzlicht, and Obhi [39], which may provide indirect evidence for an effect of power on mimicry. This study used transcranial magnetic stimulation (TMS) to show that participants who had been given a high-power prime had significantly less motor resonance when observing actions [a possible mechanism for mimicry: [10,40]), compared to participants who received a low-power prime.

Another very different possibility is that these AI effects function at a higher level, which is more dependent on social interpretation, meaning construction, and emotion regulation. One recent example of such an “interpretational” framework is the idea of mimicry as social regulation, proposed by Hess and Fischer [41]. This model emphasizes the interactive nature of the relative roles for the perceiver and displayer, especially when social power or status cues are changing. For instance, Cheng and Chartrand [42] investigated the role of status on BM by assigning participants to the role of either worker or leader and observing their BM of a confederate who they believed had been assigned the opposite role. They found that those participants who scored high in self-monitoring were more likely to imitate a confederate who had been assigned to the leader role, compared to one who had been assigned to the worker role. Ashton-James and Levordashka [43] found a similar effect, where participants who scored above average for trait narcissism showed greater BM towards a high-status researcher (PhD student) than a low-status researcher (undergraduate).

However, another line of research suggests that the rote reflection of observed behaviours is not always adaptive in hierarchical contexts. As Heyes [44] notes, the actions between a high- and low-status individual consist not of mimicry, but rather of complementary behaviours (e.g., a high-status individual adopting a more dominant expansive posture, while the low-status individual adopts a more submissive constricted posture; [45]). In line with this, Tiedens and Fragale [46] used a confederate to examine both how much participants mimicked either an expansive or constricted posture, and how the effect of mimicry and complementarity on perceived rapport. The study found that participants tended to produce complementary postures to the confederate, and they rated confederates who adopted a complementary posture as more likable (compared to those who mimicked). These findings highlight the potential limits of mimicry as “social glue,” particularly in hierarchical situations.

Further, Carr, Winkielman, and Oveis [47] used EMG to show that the FM is modulated by the power state of both the displayer and observer. More specifically, high-power observers did not smile back to smiles from high-power displayers, even though they showed a normal mimicry pattern toward low-power displayers (i.e., increased zygomaticus activity to low-power smiles). These findings suggest that simple, direct mimicry might not always be the best way to adapt to social interaction (especially in situations where hierarchical cues are made salient). In fact, from the observer’s perspective, simple, direct, and unqualified mimicry can be perceived as a sign of social or intellectual incompetence [48].

### Measuring the effects of status and power on AI

As described above, there are two key methods for measuring social modulations of motor mimicry. The first is the naturalistic measurement of BM, and the second is the SRC paradigm to measure AI. Each method has distinct advantages and disadvantages [6], especially given different contexts where they are used. The BM approach involves a richer social environment, which is potentially more informative for generalising to real-world social cognition. In contrast, the AI imitation approach offers great experimental control, as well as precise measurement of action timing. However, it functions within a more impoverished context, so when social information is present, it is usually conveyed in abstract fashion.

Here, we adopt the second approach. SRC paradigms give us full control over other environmental social cues, which could potentially interfere with effects in naturalistic paradigms [49]. Testing the effects of status and power on AI also serves as a strong and informative test for the question of the *automaticity* for any modulation of imitation, since AI task performance appears to be largely outside one’s capacity for intentional control [10]. For example, R. Cook, Bird, Lünser, Huck, & Heyes [50] found that participants tended to automatically imitate even in a competitive contexts, where imitation actually *hurt* performance outcomes. In addition, Hogeveen and Obhi [51] showed that AI is not affected by participants’ expectations about whether the trial was likely to be congruent or incongruent—another key hallmark of automaticity. Previous studies on status and mimicry [42,43,46] primarily used the BM methodology, which is only able to demonstrate automaticity where participants were unaware that they were mimicking the confederate. In contrast, using the SRC paradigm allowed us to test whether the effects of status on the tendency to mimic were automatic at a more basic level.

### The current study

The above review provides theoretical reasons both for and against the idea that status and power may modulate AI. The present paper describes a series of five experiments that sought to examine how social status and power modulate such motor mimicry of intransitive hand movements (i.e., ones that are *not* goal-directed). Note that Experiments 1–4 were conducted by HF, MS, and AH, while Experiment 5 was conducted separately by EC and PW. Both groups were independently motivated by the same question and adopted similar methods, and in bringing these results together, found that a stronger assessment of the relationship between AI, status, and power could be made.

With the following five studies, we investigated the effect of status and power on AI of intransitive finger movements by manipulating the social status (Experiments 1 and 2), competence (Experiment 3), and power (Experiments 4 and 5) of participants or their interaction partners.

## Experiment 1

### Methods

#### Design

This study assessed the impact of two actors’ social status on participants’ tendencies to mimic the actors, using an automatic imitation (AI) task. The experiment was designed as a 2 (Congruency: congruent, incongruent) x 2 (Status: high, low) within-subjects design.

The study had two phases: a status-learning phase (where participants associated high- or low-status characteristics with two different faces), followed by an AI phase. The dependant variables were reaction time measured in the AI phase, along with performance on the status-learning task. A post-experiment questionnaire served as manipulation checks for status knowledge.

#### Materials

Social status-learning task: Videos and still images of two female actors (aged approximately 23 years old) that had previously been matched for attractiveness were used to represent the two characters in this study [52]. Twenty-one statements each described a high-status person and low-status person, which were created specifically for this experiment. The high-status character was framed as a successful West End actor with items including, ‘She received great reviews from her latest acting performance’ and ‘She is admired among her colleagues.’ The low-status character was set up as an unsuccessful waitress with items including, ‘She did not get accepted for university’ and ‘She has been warned to improve her performance at work’ (see Appendix for the full list of statements). Chartrand and Bargh [1] emphasise that social group stereotypes are activated by the mere observation of distinctive features that characterize the group. Thus, such stereotypical statements should effectively enable participants to recognize the social status of each character (see also [53]).

**Fig 1:**
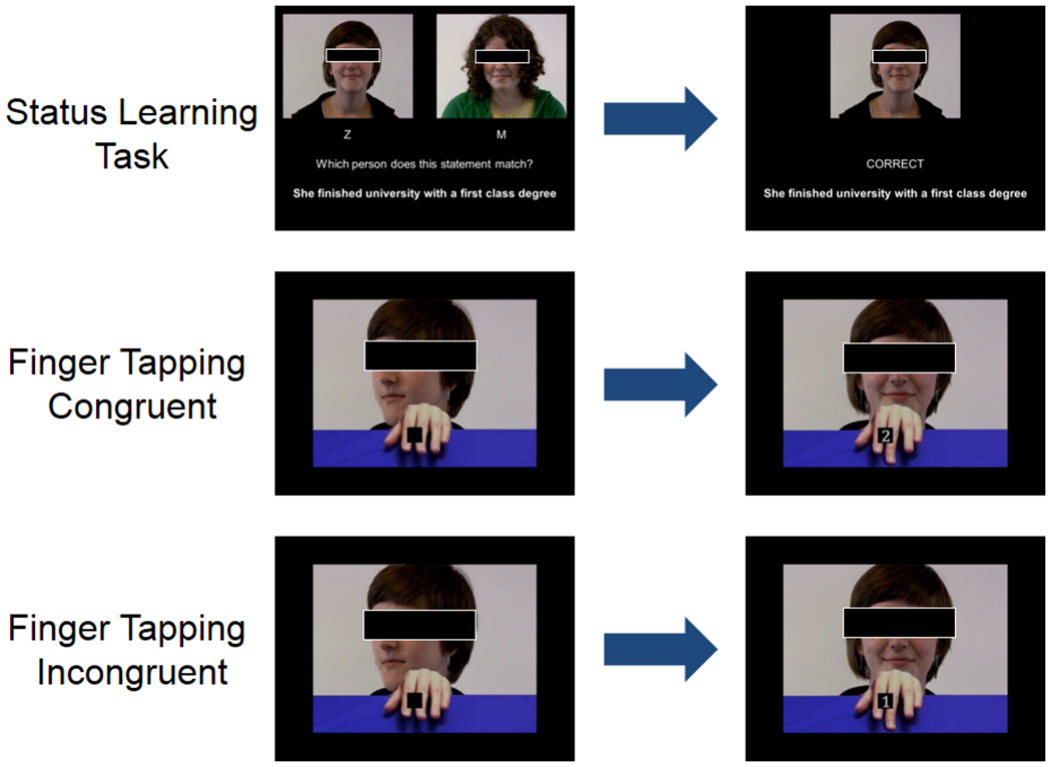
Structure of status-learning and finger-tapping tasks. Models’ faces were fully visible in original stimuli but have been occluded here in order to preserve their anonymity.

To ensure that participants engaged with the characters (and their respective status assignments), the training phase of the study used the structure of an associative learning task. On each trial, participants saw one statement (from the total set of 42 above) and the faces of the two actors on the left or right side of the screen (see Fig 1A). They were asked to decide which face matched the statement by pressing a left/right key, and feedback (correct vs. incorrect) was given after each trial. At the start of the task, participants were not given any information on which face was high- or low-status, but they were rapidly able to learn the difference. The identities of the high- and low-status actors were counterbalanced across participants.

Finger-tapping task: A computerized finger-tapping task [9] was used to measure AI. Short video clips (3–4 seconds) were prepared, showing one of the actors seen in the status-learning task resting her hand on a table in front of her. The hand image on the screen was oriented to mirror participants’ dominant right hand. In each video clip, the actor began with her face turned to the side, then turned to look directly at the participant, after which she made a finger movement with either her index or middle finger. At the same time, a number cue (1 or 2) appeared in a black box between the index and middle finger (see Fig 1B). The delay between the head turn and the finger movement/number cue varied between 1200 and 1800 milliseconds (ms), in order to avoid anticipatory responding.

Participants were instructed to respond to the number cue by pressing a key with their index finger when seeing a ‘1’ and with their middle finger when seeing a ‘2’. This meant that on some trials, the participant’s finger movement was congruent with the movement of the actor, while on other trials, the participant’s finger movement was incongruent (the instructions also emphasised the importance of fast, accurate responding). The task was presented in three blocks of 32 trials, which contained equal numbers of trials in each cell of the 2 x 2 factorial design (by Congruency and Status) in a pseudorandomised order.

Social attribute questionnaire: To test whether or not the status manipulation was successful, participants were asked to rate both characters on competence, likability, attractiveness, admiration, and social status. Each attribute was rated on a 7-point Likert scale.

#### Procedure

Participants were given a verbal briefing about the two tasks and then told to go through the program at their own pace. Both tasks were implemented in a single script using Cogent 2000 (developed by the Cogent 2000 team at the FIL and the ICN), Cogent Graphics (developed by John Romaya at the LON at the Wellcome Department of Imaging Neuroscience), and Matlab 2014b [54]. Written instructions were given before each block of trials. First, participants completed a single block of 30 status-learning trials presented in a randomised order. Half the statements characterised the high-status character, and the other half characterised the low-status character. Next, participants completed a block of 32 trials of the finger-tapping task. A status-learning reminder block of six trials was then performed, followed by a second block of 32 finger-tapping trials. A third block of six status-learning trials was carried out prior to the third and last block of 32 finger-tapping trials. This resulted in a total of 42 status-learning trials and 96 finger-tapping trials. The shorter reminder blocks of the status task were implemented in between the finger-tapping blocks to remind participants of the status for each of the two characters.

When the computerized experiment was completed, participants were asked to fill in the social attributes questionnaire for each character. Next, participants were asked a series of questions for their thoughts about the experiment, in order to gain insights about their understanding (and to ensure that the true purpose of the experiment was not revealed). Finally, participants were thoroughly debriefed and given a detailed sheet explaining the real purpose of the experiment.

#### Participants

Thirty right-handed participants gave their written informed consent to participate and were paid for their participation. Two participants were excluded, due to performing 2 standard deviations below the mean in the status-learning task (excluding trials 1–6), resulting in a final sample of twenty-eight participants (mean age ± SD: 26 ± 6 years; 8 males). The University College London, Institute of Cognitive Neuroscience Research Department’s Ethics Committee approved the study.

### Results

#### Status manipulation checks

To confirm that the status manipulation worked, we examined both the status-learning task and post-task questionnaires. In the status-learning task, participants responded with 88.3% accuracy. From trial 6 onwards, the average score was 92.3% correct (minimum 81.1%; maximum 100%), indicating that all participants included in the final analysis were able to learn the actors’ status.

Kolmogorov-Smirnov tests for normality found that post-task ratings for all attributes of both the high- and low-status actors had a non-normal distribution (*p* < .05). Therefore, Wilcoxon Signed-Ranks tests were carried out between the ratings for high- and low-status characters on each attribute. The tests revealed that participants gave the high-status character significantly higher ratings for social status, *Z* = -4.57, *p* < .001, *r* = -.863, competency, *Z* = -4.56, *p* < .001, *r* = -.862, admirability, *Z* = -3.88, *p* < .001, *r* = -.733, attractiveness, *Z* = -3.4, *p* = .001, *r* = -.642, and likability, *Z* = -2.86, *p* < .01, *r* = -.54 (see Table 1).

**Table 1:** Ratings on social attributes questionnaire for Experiment 1.

Attribute	High Status		Low Status	
	M	SD	M	SD
**Social Status**	6.25	0.93	2.00	1.05
**Competency**	6.25	0.89	2.68	1.28
**Admirability**	5.86	1.24	3.04	1.55
**Likeability**	5.46	1.48	3.90	1.21
**Attractiveness**	5.21	0.92	4.00	1.15

#### AI

Reaction time (RT) was the primary outcome measure in the finger-tapping task. Incorrect responses (3.59%) were excluded from the finger-tapping task analysis, as were all RTs less than 200 ms or greater 1000 ms (0.83%). Additionally, the first trial of each block functioned as a practice trial, which was eliminated from the analysis.

**Fig 2:**
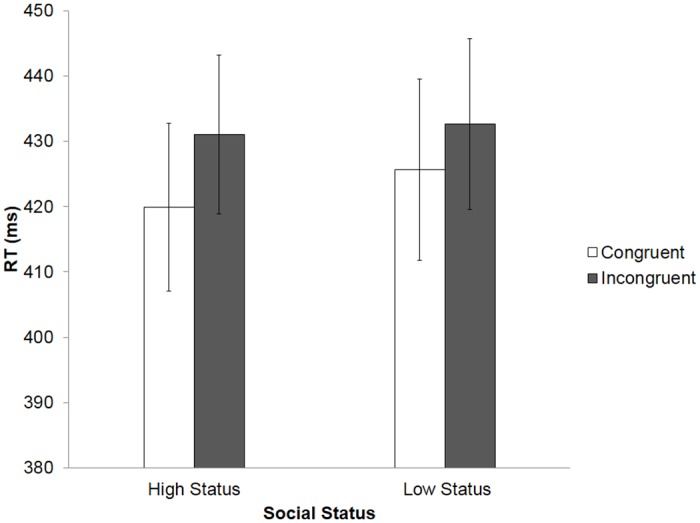
Reaction times (RTs) across all conditions of the finger-tapping task in Experiment 1. Error bars represent standard error of the mean (SEM).

A repeated measures ANOVA on RT data uncovered a significant main effect of Congruency, *F*(1, 27) = 5.58, *p* = .026, η^2^ = .171, with a faster response to congruent trials (M = 422 ms, SD = 70 ms) and a slower response to incongruent trials (M = 431 ms, SD = 66 ms). However, we observed no Status effect on RTs, *F*(1, 27) = 1.46, *p* = .238, η^2^ = .051, and no significant Congruency x Status interaction, *F*(1, 27) = 0.30, *p* = 0.590, η^2^ = .011 (see Fig 2).

### Discussion

Experiment 1 revealed a clear congruency effect during the finger-tapping task, with faster responses on congruent trials—which replicates many previous studies using this task [4,55]. The analysis of both the status-learning task and social attributes questionnaire suggested that participants learned the status of the two actors and discriminated between them on the basis of status. However, there was no evidence that participants mimicked the high-status actor more than the low-status actor.

One possible explanation for this null result is that in typical social interactions, we do not switch rapidly between different interaction partners but instead remain engaged with a single person for extended periods of time [7]. During Experiment 1, the trial order was fully randomised, with the high- and low-status characters appearing in an unstructured order. This requirement to rapidly switch between people might disrupt any potential social modulation of AI.

In order to test this explanation, we conducted Experiment 2. With Experiment 2, we made a simple change to the structure of the finger-tapping task. Instead of mixing the high- and low-status actors within a single block, the characters were presented in separate blocks. This ensured that participants had ample opportunity to adjust to the character for that particular block. We anticipated that this simple change to the design would more accurately resemble a real-life interaction.

## Experiment 2

### Method

#### Design, Materials, and Procedure

The stimuli, design, and procedure were identical to Experiment 1, except for the change in trial ordering.

For Experiment 2, participants first performed 24 status-learning trials, followed by 24 AI trials. Next, participants completed three more sets of trials, each consisting of six top-up status trials, followed by another 24 AI trials. This gives a total of 42 status-learning trials and 96 AI trials, per participant. Within each set of 24 AI trials, the first 12 showed one character, and the second 12 showed the other character. The order of characters and status-levels were counterbalanced across participants.

#### Participants

Twenty-five right-handed participants gave their written informed consent to participate and were paid for their participation. One participant was excluded because they accurately guessed the hypothesis of the study, and another was excluded due to performing 2 standard deviations below the mean in the status-learning task (excluding trials 1–6), resulting in a final sample of twenty-three participants (mean age ± SD: 24 ± 4 years; 9 males). The University College London, Institute of Cognitive Neuroscience Research Department’s Ethics Committee approved the study.

### Results

#### Status manipulation checks

In the status-learning task, participants responded with 84.9% accuracy, and from trial number 6 onwards, the percentage of correct responses increased to 89.2% (minimum 75.7%; maximum 97.3%), indicating that the status-learning task was successful.

Kolmogorov-Smirnov tests for normality found that post-task ratings for all attributes of both the high- and low-status actors had a non-normal distribution (*p* < .05). Therefore, Wilcoxon Signed-Ranks tests were carried out between the ratings for high- and low-status characters on each attribute. The tests revealed that participants gave the high-status character significantly higher ratings for social status, *Z* = -3.93, *p* < .001, *r* = -.819, competency, *Z* = 3.76, *p* < .001, *r* = -.785, and admirability, *Z* = -3.55, *p* < .001, *r* = -.74. Ratings of likability were also marginally significantly higher for the high-status character, *Z* = 2.11, *p* = .067, *r* = -.381. However, no significant effect of Status was found for ratings of attractiveness, *Z* = -1.2, *p* = .232, *r* = -.249 (see Table 2).

**Table 2:** Ratings on social attributes questionnaire for Experiment 2.

Attribute	High Status		Low Status	
	M	SD	M	SD
**Social Status**	6.09	1.04	2.35	1.30
**Competency**	6.22	0.95	3.17	1.27
**Admirability**	5.78	1.28	3.26	1.29
**Likeability**	5.43	0.79	4.74	1.18
**Attractiveness**	4.48	1.20	4.09	1.04

#### AI

Incorrect responses (3.29%) were excluded from the finger-tapping task analysis, as were all RTs less than 200 ms or greater 1000 ms (0.33%). Additionally, the first trial of each block functioned as a practice trial, which was eliminated from the analysis.

**Fig 3:**
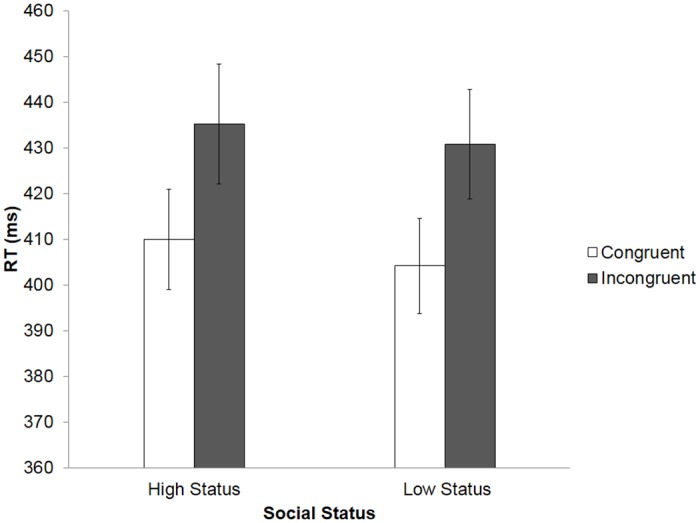
Reaction times (RTs) across all conditions of the finger-tapping task in Experiment 2. Error bars represent standard error of the mean (SEM).

A repeated measures ANOVA on RT data demonstrated a significant main effect of Congruency, *F*(1, 22) = 26.97, *p* < 0.001, η^2^ = .551, with faster RTs to congruent trials (M = 407 ms, SD = 50 ms) and slower RTs to incongruent trials (M = 433 ms, SD = 60 ms). As in Experiment 1, the ANOVA found no effect of Status on RTs, *F*(1, 22) = 1.37, *p* = .255, η^2^ = .059, and no significant interaction between Congruency and Status, *F*(1, 22) = 0.38, *p* = .847, η^2^ = .002 (see Fig 3).

### Discussion

Experiment 2 replicated Experiment 1 with clear evidence of status-learning in the post-task questionnaires and a clear congruency effect in the AI task. As before, there was no Congruency x Status interaction, meaning that the social status of the character did not modulate AI (as measured in the finger-tapping task). This suggests that the failure to find an effect of status in Experiment 1 was not merely an artefact of trial ordering. Taken together, the results of Experiments 1 and 2 suggest that simply being aware of the difference in social status between two people does not lead to any modulation of the tendency to mimic those people.

Note that in Experiments 1 and 2, social status was manipulated by creating one character with a high social status, while the other had a low social status. However, this did not require participants to compare the status of the two characters with their own status. In contrast, previous studies investigating AI and status [42,43] directly manipulated the status of the character to be mimicked relative to the participant. Cheng and Chartrand [42] assigned the mimicker to be either a worker or leader (relative to their confederate), while Ashton-James and Levordashka [43] manipulated whether the confederate was presented as a PhD student or an undergraduate (a highly relevant status distinction for university students). Previous studies [4,56] have demonstrated that the self-relevance of a social prime can change how that prime affects AI. Therefore, it is possible that the lack of self-relevance in the status manipulations for Experiments 1 and 2 could have led to a null result.

To examine this possibility, Experiment 3 manipulated the status of the two characters via competence, using a letter-game paradigm (see [57] for a similar manipulation). Fiske, Cuddy, Glick, and Xu [58] demonstrated that perceptions of competence and status are highly correlated, both for social group stereotypes and individual-based judgments. Therefore, we predicted that participants would judge a character that did better at the letter game as having a higher status than a character that did worse. Manipulating competence in the letter-game task allowed us to encourage participants to compare their own status to that of the characters, by having the participants also take part in the game (thus always performing better than one of the characters, and worse than the other).

## Experiment 3

### Methods

#### Design

With Experiment 3, we aimed to test whether status modulates AI when the status of a character is manipulated relative to the status of the participant in a competitive game. The experiment was designed as a 3 (Congruency: congruent, incongruent, baseline) x 2 (Status: high, low) within-subjects design. The dependent variables were reaction time (RTs) in the finger-tapping task and questionnaires about the characters (which served as a manipulation check).

#### Materials

The experiment was presented to participants on a 15-inch Lenovo laptop screen (60hz refresh rate). Both the letter game and the finger-tapping tasks were implemented in Vizard 4.10 [59]—a virtual reality software package, which allowed us to create virtual characters (VCs) and control their social behaviour. Using virtual reality provides a more realistic and interactive social experience, while retaining high experimental control.

Two female VCs were chosen for this study, with distinct voices and appearance. One VC was assigned high-status, and the other was assigned low-status. One was consistently seen on the left of the screen, with the other on the right. Both of these factors were counterbalanced across participants.

**Fig 4:**
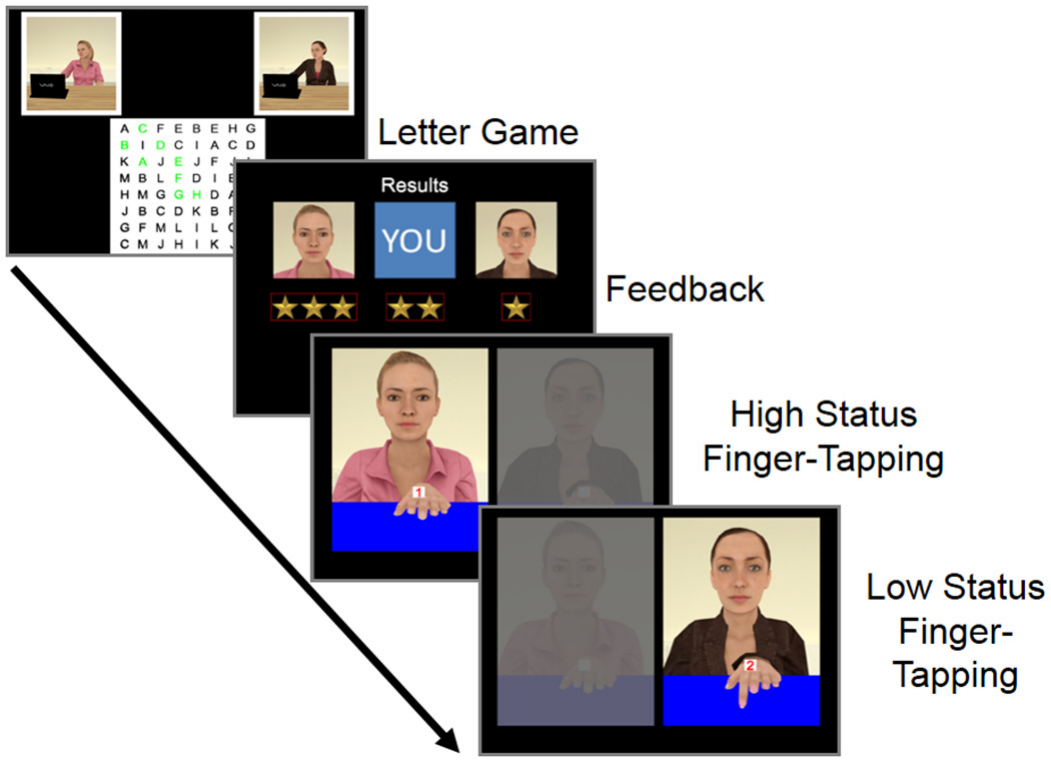
Sequence of tasks for Experiment 3.

Letter-game task: Status was primed using a grid task in which participants saw an 8 x 8 grid of letters, and they had to select letters in order to navigate a path from ‘A’ to ‘M’. They were instructed to complete the grid as fast as possible, in competition with the two VCs who were visible in separate windows located in the left and right hand corners of the screen (see Fig 4). The task was set up such that the high-status VC would always complete the grid when the participant was between 1 and 4 letters from completion, whereas the low-status VC would always complete the grid 1.5 to 4 seconds after the participant finished. As each VC completed the grid, they would look up from the computer and announce ‘*Done*’ in either a proud (high-status) or disappointed (low-status) tone of voice. The border of the VCs window also turned green on completion, and the border of the letter grid turned green when the participant completed the grid. When all players (participant plus two VCs) were done, participants saw a scoreboard indicating their position (always 2^nd^ place), compared to the position of the two VCs.

Finger-tapping task: AI was measured using a modified version of the finger-tapping task from Experiments 1 and 2. The two VCs were placed on the left and right of the screen consistent with their previous locations, behind semi-opaque grey screens. Each VC held her left hand just below her face, mirroring the participant’s right hand in a similar configuration to Experiments 1 and 2. Both VCs were present in all trials, in order to highlight the contrast between their performances in the letter game.

During each trial, the grey screen vanished from one VC, and after a variable delay of one to two seconds, participants saw a numeric movement cue together with a finger movement from the VC. These finger movements were generated using key frame animation in MotionBuilder 14.0 [60]. The associations between the numeric cue and finger movements were the same as in Experiments 1 and 2, but a new baseline condition was added in which a cue appeared, but the VC did not execute a finger movement. The inclusion of a baseline condition allowed the direction of any congruency effect modulation induced by status to be determined.

As in Experiment 2, the VCs were presented in blocks of 60 trials, with the first 30 trials of each block consistently displaying one VC, and the remaining 30 trials of each block displaying the other VC. In each set of 30 trials, 12 were congruent, 12 were incongruent, and six were baseline. The order of these 30 trials was fully randomised. In addition, each block started with one extra baseline trial which was discarded in the analysis. RTs were recorded for a total of 240 trials (four blocks of 60 trials).

Manipulation checks: In order to check that our status manipulation was effective, participants were asked to complete two computerized questionnaires, each asking for their feelings about one of the VCs.

They were shown 12 statements, which were grouped into four subscales of three questions each. The first three subscales measured participants’ view of the VC’s intelligence, competence, and social economic status (SES), while the final subscale measured the perceived rapport between the participant and the VC (see Table 3). For each statement, participants were asked to indicate how much they agreed or disagreed by placing a marker along a slider. The slider’s values ranged from 0 to 1, and the marker was randomly positioned between 0.4 and 0.6 at the start of each question.

**Table 3:** Manipulation check statements used in Experiments 3 and 4 (* indicates reverse scoring).

Statement	Subscale	Experiment
I felt that Beth seemed responsible.	Competence	3 & 4
I thought that Beth was efficient.	Competence	3 & 4
I felt that Beth seemed inept. *	Competence	3 & 4
I thought that Beth seemed bright.	Intelligence	3 & 4
I felt that Beth was intelligent.	Intelligence	3 & 4
I thought that Beth was stupid. *	Intelligence	3 & 4
I think that Beth has a prestigious job.	SES	3 & 4
I felt that Beth seemed well educated.	SES	3 & 4
I felt that Beth was economically unsuccessful. *	SES	3 & 4
I felt like I was in tune with Beth.	Rapport	3 & 4
I would go for coffee with Beth.	Rapport	3 & 4
I felt positive about Beth.	Rapport	3 & 4
I felt that my wishes didn’t carry much weight with Beth.	Power	4
I was able to get my way in the card game with Beth. *	Power	4
Beth had a great deal of power in the card game.	Power	4
I had an influence on decisions in the card game with Beth. *	Power	4
Beth was not in control of the card game. *	Power	4
Beth led the card game.	Power	4

#### Procedure

Participants were given a verbal briefing about the two tasks and then told to go through the program at their own pace. Written instructions were given on the screen before each task. Participants were first introduced to each of the VCs and then completed three trials of the letter-game task, to ensure they understood that the performance of the two VCs was not due to chance.

Next, they completed one block of the finger-tapping task. Participants then did three blocks of the finger-tapping task, each of which was preceded by one trial of the letter-game task. Once all blocks were completed, participants answered the questionnaire for each VC with the order of presentation counterbalanced across participants. Finally, participants were debriefed about the purpose of the experiment.

#### Participants

Twenty-two right-handed participants (mean age ± SD: 22 ± 3 years; 10 males) gave their written informed consent to participate and were paid for their participation. The University College London, Institute of Cognitive Neuroscience Research Department’s Ethics Committee approved the study.

### Results

#### Manipulation check

For the manipulation check, participants’ responses to the three statements in each of the four subscales were each averaged together to give a mean response for each subscale.

Kolmogorov-Smirnov tests for normality found that the competence subscale for the low-status character had a non-normal distribution (*p* < .05), while all other subscales had a normal distribution (*p* > .05). In order to check that our letter-game manipulation did indeed affect participants’ judgements about the VCs’ status, paired sample *t*-tests were carried out on each the averaged responses for the intelligence, SES, and rapport subscales (see Table 4). A Wilcoxon Signed-Ranks test was carried out on the competence subscale. The tests revealed that participants rated the high-status VC as significantly more efficient, *Z* = -3.49, *p* < .001, *r* = -.744, intelligent, *t*(21) = 5.1, *p* < .001, *r* = .743, and as having a higher SES, *t*(21) = 4.35, *p* < .001, *r* = .690, than the low-status VC. In contrast, no significant difference was found between the high- and low-status VC for rapport, *t*(21) = -1.62, *p* = .121, *r* = .323.

**Table 4:** Means and SDs for questionnaire subscales in Experiment 3.

Subscale	High Status VC		Low Status VC	
	M	SD	M	SD
**Competence**	0.71	0.14	0.51	0.12
**Intelligence**	0.75	0.13	0.49	0.16
**SES**	0.71	0.14	0.5	0.15
**Rapport**	0.44	0.2	0.53	0.17

#### AI

As in the previous experiments, incorrect responses (3.10%) were excluded from the finger-tapping task analysis, as were all RTs smaller than 200 ms or greater 1000 ms (0.80%).

**Fig 5:**
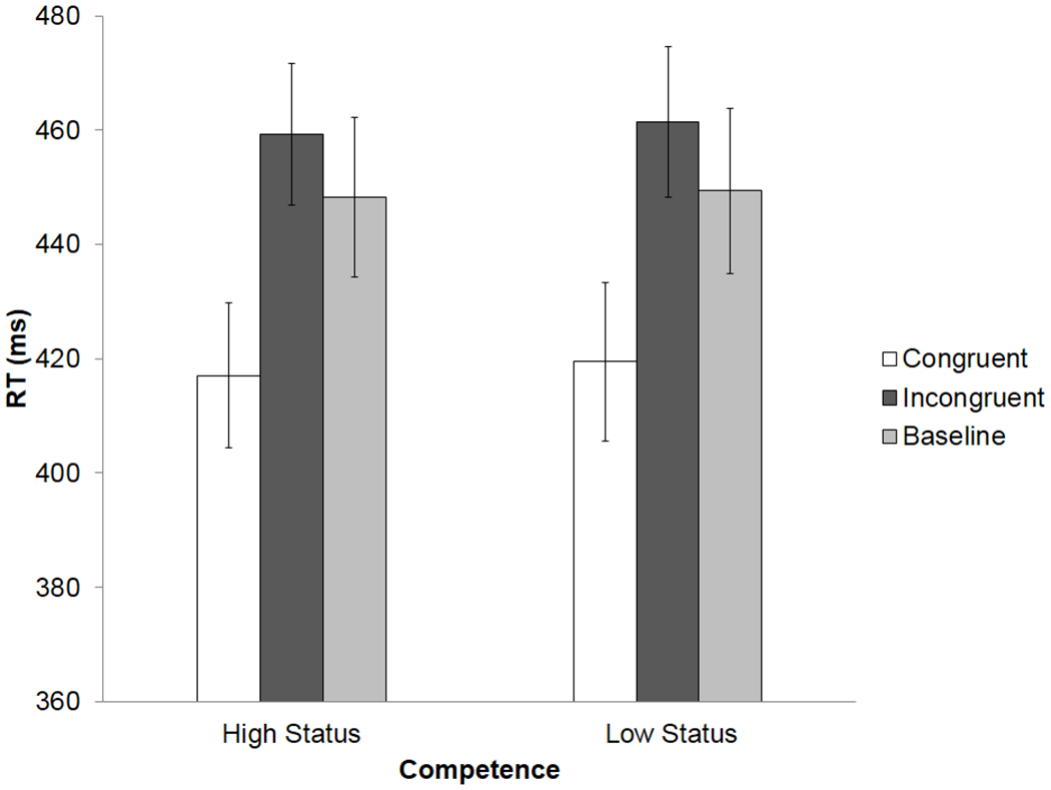
Reaction times across all conditions of the finger-tapping task in Experiment 3. Error bars represent standard error of the mean (SEM).

In order to examine whether the status of the VC had influenced AI, a 2 x 3 repeated measures ANOVA was carried out on the RT data, which included the factors of Congruency (3: congruent, incongruent and baseline) and Status (2: high, low) (see Fig 5). The ANOVA revealed a significant effect of Congruency, *F*(2, 42) = 5.58, *p* < .001, η^2^ = .714, with congruent trials resulting in the faster RTs (M = 418 ms, SD = 62 ms) and incongruent trials resulting in the slower RTs (M = 460 ms, SD = 59 ms), while baseline trial responses were intermediate (M = 449 ms, SD = 66 ms). However, there was no significant effect of Status, *F*(1, 21) = 0.67, *p* = .422, η^2^ = .031, nor was there a significant Congruency x Status interaction, *F*(2, 42) = 0.02, *p* = .982, η^2^ = .002.

### Discussion

The aim of Experiment 3 was to examine whether emphasizing the relative difference in status between one’s self and another character would lead to a modulation of AI by status. Participants’ responses to the post-questionnaire measures confirmed that the letter game successfully manipulated all three of the status subscales used (i.e., the VC who performed better than the participant was rated as higher in social status, competence, and intelligence than the VC who performed worse than the participant). Moreover, the study showed a strong congruency effect, even though the models used were virtual characters (rather than actual people). However, despite the successful status manipulation, there was no effect of status on participants’ tendency to mimic the VC. The results of this study further support the findings of Experiments 1 and 2—even when the status of the observed character is directly contrasted with that of the participant, status does not have a modulating effect on AI.

One possible confound of the current study is that while the responses to the post-study questionnaire shows that the letter-game manipulation did indeed manipulate status, it also involved a competitive setting where the high-status character outperformed the participant. Thus, it is possible that any effect of status in this study could have been masked by a corresponding contrary effect of competition. However, against this possibility, previous studies have shown that competition leads to greater overlap in motor behaviour [61], and people tend to imitate others even in competitive situations where imitation is actively opposed to their strategic interests [50,62].

In light of these findings, we shifted our research to focus more on power. As discussed in the introduction, although status and power are closely related constructs, they are not identical. While social status is based on the amount of social respect and admiration, social power depends more on an individual’s ability to control others’ access to valued resources. If AI is truly used in a “Machiavellian” manner, it may be more likely to be modulated by the actual power an individual holds in a situation, rather than by their general social status.

We investigated this idea in Experiment 4, using VCs that varied across three different power levels: high power (where the VC could make choices that affected the monetary pay-out the participant would receive), low power (where the participant could make choices that affected the monetary pay-out the VC would receive), and neutral (where the participant made choices affecting his/her own monetary pay-out). In addition, we sought to expand on the findings from Cheng and Chartrand [42] by investigating whether self-monitoring plays a mediating role between power and AI.

## Experiment 4

### Methods

#### Design

The experiment was designed as a 2 (Congruency: congruent, incongruent) x 3 (Power: high, low, neutral) within-subjects design. The dependent variables were AI measured from RTs, and participants’ agreement with a series of statements (which served as a manipulation check for the power prime).

#### Materials

The experiment was presented to participants on a 15-inch Lenovo laptop screen (60hz refresh rate) implemented in Vizard 4.10, using the same two VCs as Experiment 3.

Choice task: Power was primed using a modified version of the choice task developed by Leotti and Delgado [63]. There were three variants of the task giving different levels of power to the VC (high-power, low-power, and neutral power). In the choice task, participants saw an orange and a purple card on the screen, one on the left and one on the right. Participants were told that the different cards had different reward probabilities (£0, £50, £100) and that they would be paid according to the reward they won when the cards were chosen. They were also informed that the value of each card could change during the study. In actuality, the reward generated by each choice was randomly assigned from a set that contained an equal number of each outcome such that, over the entire length of the choice task, all participants received the same amount of reward for each condition. This equal distribution of reward ensured that any effect of condition on AI would be due to the minimal power involved in choosing a card, rather than being due to the participant receiving greater objective rewards when making choices themselves as opposed to receiving the choices of the VC.

**Fig 6:**
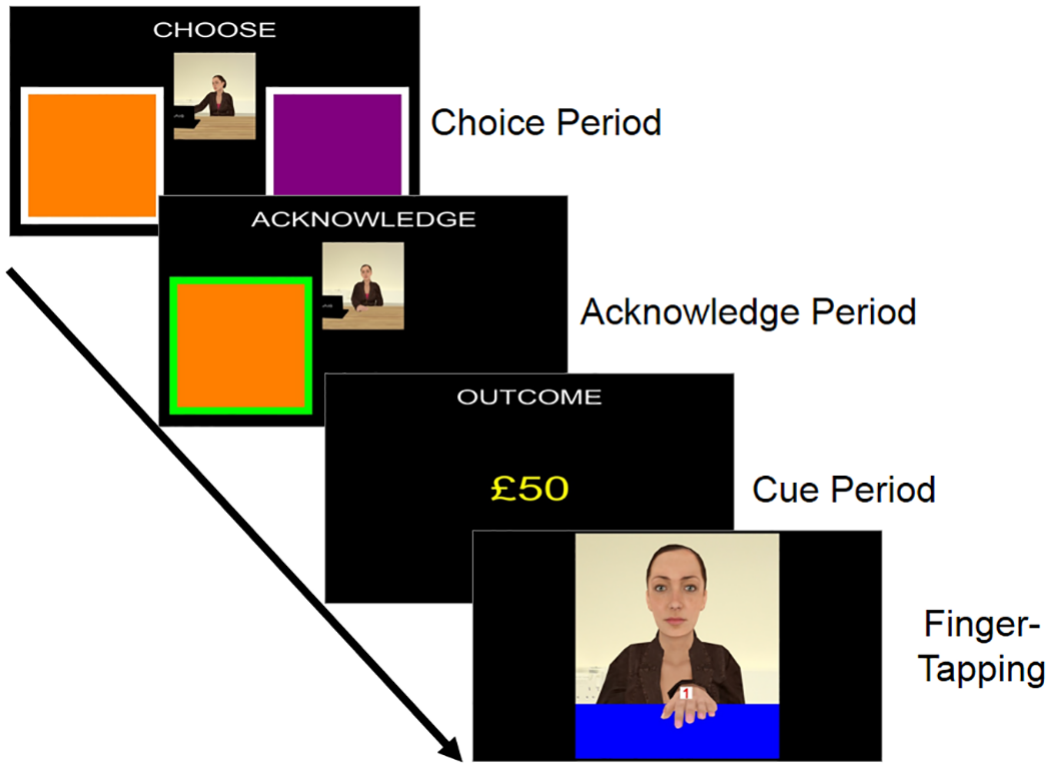
Sequence for choice trial and example of finger-tapping trial in Experiment 4.

In all three versions of the choice task, each trial had three key stages (each with a two-second duration). First, during the choice stage, the participant (or VC) had to select one of the two cards by pressing either a left or right key on the keyboard; after selection, the chosen card was highlighted in green. Second, during the acknowledge stage, the card that was *not* selected disappeared from the screen, and the participant (or VC) had to acknowledge the previous choice by pressing the key corresponding to the remaining card. Once pressed, a green star appeared on the screen for the remainder of the two seconds. Finally, the outcome stage stated one of the three reward amounts (to represent the reward for the selected card). Failure to choose or acknowledge a card led to an error trial, in which no points were received (see Fig 6).

The three versions of the task varied, according to who responded during each round. With the high-power VC version, a single VC was visible in the centre of the screen. Participants were told this person would make the choice, and that they would have to acknowledge the choice in order to receive the points. The VC looked at the keyboard when making her choice (and at the participant during the acknowledgments). With the low-power VC version, the other VC was visible on screen. Participants were told that they would choose the card and that the VC would acknowledge the choice before receiving the reward (and the VC’s behaviour matched this sequence). With the neutral power variant of the task, no VC was visible. Participants were told that they would choose the card, acknowledge the outcome, and receive the reward. As before, the visual appearance and voices of the high-power and low-power VCs were constant across rounds for each participant (but counterbalanced across participants). In order to ensure that participants felt they were making real decisions and interacting with real people during the task, they were told that the decisions made by the high-power VC reflected decisions made by a previous participant, while decisions made by the participant for the low-power VC would be imposed on a future participant.

The choice task was presented in a blocked format, with each block of the task consisting of six trials. At the beginning of the block, participants were informed who would be making the choice and who would be gaining the reward. At the end, they were informed of the number of valid choices made and the number of points earned.

Finger-tapping task: AI was measured using the finger-tapping paradigm from Experiment 3, with three modifications. First, a neutral version of the task was developed, where instead of seeing a VC on the screen, participants only saw a hand on a blue background. Second, the baseline trials were removed, as the inclusion of the neutral condition made them unnecessary (i.e., each block of AI consisted of 32 randomised trials—16 congruent and 16 incongruent). Third, only the VC relevant to the current block was visible in the centre of the screen.

#### Procedure

Participants were given a verbal briefing about the two tasks and then told to go through the program at their own pace. To ensure participants understood the task, written instructions were given on the screen before each block. Participants were first introduced to each of the VCs and completed 12 practice trials for each variant of the choice task, in order to ensure that they understood all task instructions. The same VCs with the same task rules were used in the practice trials and the main study, so the practice trials provided an initial opportunity to learn the VCs’ power role.

For the main study, participants completed nine sets of trials, where each set comprised of a block of six choice trials with one VC (high-power // low-power // neutral) followed by a set of 32 AI trials with the same VC (high-power // low-power // neutral). The sets were ordered such that all three power conditions were represented every three sets, but the same set never appeared twice in a row. In order to check that the power manipulation was successful, a probe question was given to participants after every set of trials. The question was taken from Guinote [64,65], which asked participants ‘*How in charge of the situation did you feel in the last block*?’ Participants had to indicate the amount of control they felt by moving a marker along a slider that went from 0 (*not in charge at all*) to 1 (*very much in charge*). The marker was randomly positioned between 0.4 and 0.6 at the start of each question. Participants were informed at the start of the experiment that these questions would always refer to the choice task, rather than the AI task.

Following the completion of all nine sets of trials, participants answered two questionnaires about the VCs, and the order of presentation was counterbalanced across participants. These included the same 12 statements that were presented in Experiment 3, and they also included an additional six questions relating to how powerful they perceived the VC during the choice task (adapted from a questionnaire used by J. E. Cook, Arrow, & Malle, [66]). Finally, participants were debriefed about the purpose of the experiment and informed of the fact that there was no systematic relationship between their choice of cards and the reward. Also, rather than being paid based on performance, all participants were paid at a flat rate of £7.50 per hour.

#### Participants

Thirty-one right-handed female participants (mean age ± SD: 22 ± 4 years) gave their written informed consent to participate and were paid for their participation. This experiment only tested female participants, and only female VCs were used, due to the existence of large gender differences in the perception of power (e.g. [67–69]**)**. The University College London, Institute of Cognitive Neuroscience Research Department’s Ethics Committee approved the study.

### Results

#### Manipulation check

In order to check that our choice task manipulation did indeed affect participants’ judgements about their power (compared to that of the VC), a repeated measures ANOVA was carried out on participant’s responses to the probe question, for each of the three power conditions.

The ANOVA found a main effect of Power, *F*(2, 60) = 4.57, *p* = .014, η^2^ = .132, with the probe following the high-power VC resulting in the highest power rating (M = 0.64, SD = 0.27) and the low-power VC resulting in the lowest power rating (M = 0.47, SD = 0.3). The response for the neutral-power probe was intermediate (M = 0.57, SD = 0.27).

Kolmogorov-Smirnov tests for normality found that the competence subscale for the high-status character and the power subscale for the low-status character both had a non-normal distribution (*p* < .05), while all other subscales had a normal distribution (*p* > .05). To further investigate the strength of our manipulation paired sample *t*-tests were carried out on each the averaged responses for the intelligence, SES, and rapport subscales, and Wilcoxon Signed-Ranks tests were carried out on the competence and power subscales (see Table 5). The tests found no significant differences in participants’ ratings of the VCs’ competence, *Z* = -0.96, *p* = .337, *r* = -.172, intelligence, *t*(30) = 0.34, *p* = .73, *r* = .052, social economic status, *t*(30) = 1.13, *p* = .27, *r* = .201, or rapport, *t*(30) = -1.48, *p* = .15, *r* = .261. However, there was a significant difference between participants’ ratings of power, with the high-power VC rated as more powerful than the low-power VC, *Z* = -2.58, *p* = .01, *r* = -.463.

**Table 5:** Means and SDs for questionnaire subscales in Experiment 4.

Subscale	Other-Choice VC		Choice-for- Other VC	
	M	SD	M	SD
**Competence**	0.58	0.15	0.55	0.11
**Intelligence**	0.6	0.15	0.58	0.17
**SES**	0.58	0.14	0.55	0.16
**Rapport**	0.51	0.18	0.57	0.15
**Power**	0.55	0.11	0.42	0.13

#### AI

**Fig 7:**
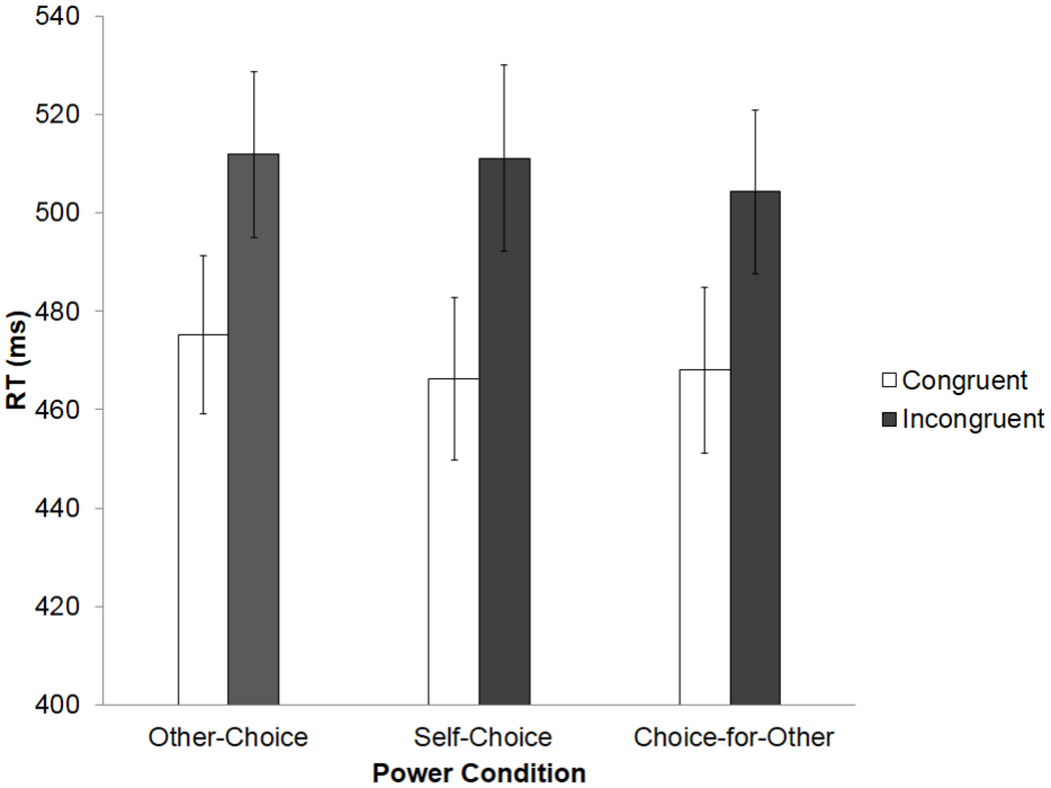
Reaction times (RTs) across all conditions of the finger-tapping task in Experiment 4. Error bars represent standard error of the mean (SEM).

As in the previous experiments, incorrect responses were removed from the analysis. Incorrect responses (2.60%) were excluded, as were all RTs smaller than 200 ms or greater 1000 ms (2.70%). In order to examine whether the power of the VC influenced AI, a repeated measures ANOVA was carried out with Power (3: high, low, neutral) and Congruency (2: congruent, incongruent) (see Fig 7).

The ANOVA revealed a significant effect of Congruency, *F*(1, 60) = 75.97, *p* < .001, η^2^ = .717, with congruent trials resulting in faster RTs (M = 470 ms, SD = 90) than incongruent trials (M = 509 ms, SD = 96). However, no significant effect was found for Power, *F*(1, 60) = 0.17, *p* = .17, η^2^ = .153, and there was no significant interaction between Congruency and Power, *F*(2, 60) = 0.06, *p* = .14, η^2^ = .108.

In order to evaluate whether participants’ levels of self-monitoring modulated the amount of AI they displayed towards the high-power VC, the scores from the self-monitoring questionnaire were normalised to the grand mean and added as a covariate in a 3 x 2 repeated measures ANCOVA, with Power and Congruency as factors. The ANCOVA found a significant main effect of Congruency, *F*(1, 58) = 73.7, *p* < .001, η^2^ = .718. We did not find any main effect of Power, *F*(2, 58) = 2.04, *p* = .1, η^2^ = .154, nor any significant interaction between Power and Congruency, *F*(2, 58) = 0.14, *p* = .2, η^2^ = .108. There was no main effect of Self-Monitoring, nor were there any interactions between Self-Monitoring and any other factor.

### Discussion

Experiment 4 investigated whether participants were more likely to imitate a VC that had power, compared to one that did not. As with the previous experiments, a strong congruency effect was found, and questionnaire responses indicated that our manipulation had the desired effect on participants’ views of the power relationship between themselves and the two VCs. However, despite the success of our manipulation, no effect of power was found in the finger-tapping task, suggesting that power does not modulate AI.

Two further considerations could possibly limit our interpretation of the results from Experiments 1–4. First, these studies used a within-subjects design, where participants experienced higher status or power than one VC, but lower status or power than another. This is in contrast to previous studies of status and AI, where between-subjects designs are often used [42,43]. Second, Experiment 4 did not actively manipulate the subjective power of the subject in a way that was independent from the stimulus—the power manipulation was inherently tied to the power level of the actor. To remedy this, in Experiment 5, we used a between-subjects design to manipulate participants’ *own* feelings of subjective power.

## Experiment 5

### Methods

#### Design

Experiment 5 used a between-subjects design and a slightly different AI task. The experiment was designed as a 2 (Congruency: congruent, incongruent) x 2 (Trial Type: movement, effector-priming) x 3 (Power: high power, low power, neutral) mixed design, with Congruency and Trial Type being within-subjects factors, and Power as between-subjects. The dependent variables were AI measured from RTs.

#### Materials

The experimental tasks were presented on a 17-inch Dell flatscreen (60hz refresh rate) using E-Prime 2.0 [70] and Visual Studio 2008 [71].

Power-priming: Power was primed by randomly assigning participants to complete either a 10-minute high-power (HP; *n*_*HP*_ = 56), low-power (LP; *n*_*LP*_ = 60), or neutral (*n*_*control*_ = 30) writing prime. Participants received instructions for the writing prime, which has been shown to reliably induce different levels of subjective power by requiring participants to recall and describe a past experience [72].

Instructions for the LP condition were as follows:

*On the sheet of paper you were given*, *please recall a particular incident in which another individual (or individuals) had power over you*. *By power*, *we mean a situation in which another person (or persons) controlled your ability get something you wanted*, *or were in a position to evaluate you*. *Write about and describe the situation—i*.*e*., *what happened*, *how it made you feel*, *etc*. *…*

Instructions for the HP condition were as follows:

*On the sheet of paper you were given*, *please recall a particular incident in which you had power over another individual (or individuals)*. *By power*, *we mean a situation in which you controlled another person (or persons) ability to get something they wanted*, *or were in a position to evaluate them*. *Write about and describe the situation—i*.*e*., *what happened*, *how it made you feel*, *etc*. *…*

Instructions for the neutral condition were as follows:

*On the sheet of paper you were given*, *please recall the timeline of events that occurred for you yesterday*. *By events*, *we mean classes*, *meetings*, *appointments*, *etc*. *Write about and briefly describe each event—i*.*e*., *what happened*, *specific times*, *etc*. *…*

**Fig 8:**
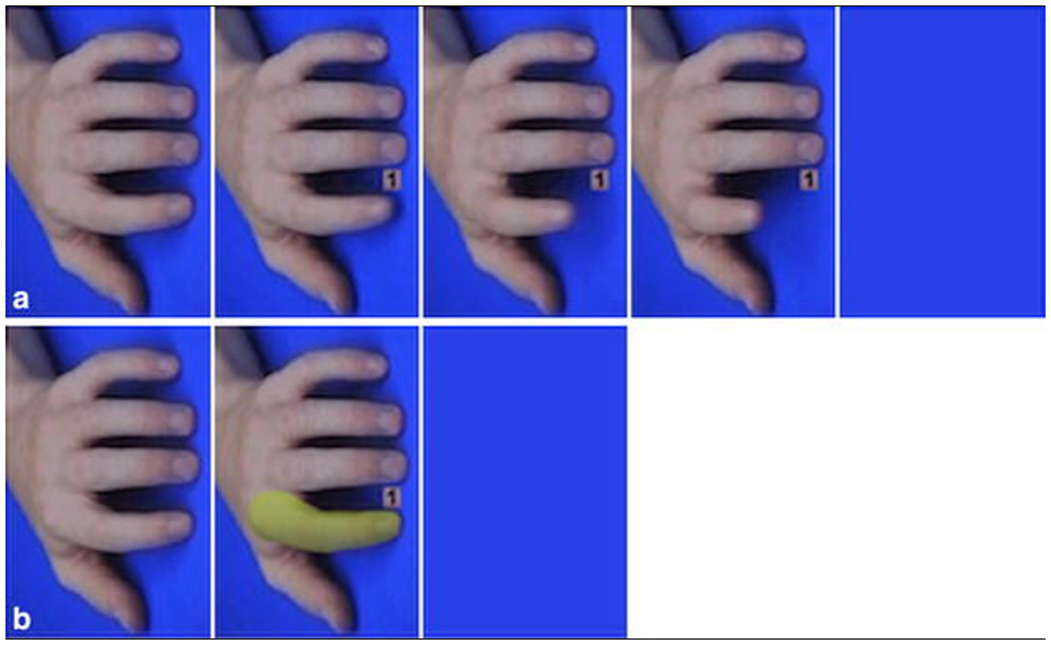
Automatic imitation task [figure and caption referenced from 73]). (a) Movement trials depicted a five-frame action video clip displayed frame 1 for a variable interval (800 ms—2,400 ms). Frames 2 and 3 were displayed for 34 ms each, and frame 4 remained on the screen until subjects responded. These display durations ensured the appearance of a short video clip. Frame 5 (a blank, blue screen) appeared on the screen once subjects logged their response. (b) Effector priming trials depicted three frames of the action video clip. Frame 1 was displayed for a variable interval (800–2,400 ms), frame 2 was displayed until subjects responded, and frame 3 (a blank, blue screen) appeared on the screen once subjects logged their response.

Finger-lifting task: AI was measured using a finger-lifting task, based on previous research (for additional details, see [73] and Fig 8). All videos depicted a human hand that was presented vertically on the screen (6° vertical visual angle x 9° horizontal visual angle), to control for spatial compatibility effects. Participants rested their right hand in a horizontal orientation (relative to the presentation screen) with their index finger on the ‘v’ key and middle finger on the ‘b’ key. At the start of each trial, participants were prompted to hold down the ‘v’ and ‘b’ keys (using their index and middle fingers, respectively), which triggered the presentation of the baseline stimulus (i.e., resting hand) that lasted for a jittered movement delay between 800 ms—2,400 ms. Following this delay, the hand in the video showed either an index or middle finger lift on movement trials (or either an index or middle finger colour prime on base trials; see Fig 8), along with either a ‘1’ or ‘2’. To respond, participants were required to lift either their own index finger (i.e., lift off the ‘v’ key, in response to a ‘1’) or middle finger (i.e., lift off the ‘b’ key, in response to a ‘2’).

50% of trials displayed a concurrent finger-lifting action that was either congruent (e.g., participants were required to lift their own index finger while observing an index finger action with a ‘1’) or incongruent (e.g., participants were required to lift their own index finger while observing an middle finger action with a ‘1’) (see Fig 8). The other 50% of trials were effector-priming trials in which the finger in the video changed colour but did not move. This was to isolate the influence of action kinematics as opposed to visual attention on automatic imitation. RTs were calculated between the last video frame presentation and participants’ key responses (i.e., lifting off the ‘v’ or ‘b’ key) on 120–128 trials. The precise number of trials varied based on performance.

#### Procedure

After signing consent forms, participants completed the writing prime task. Before data acquisition, participants received standardized instructions and completed practice trials to get them used to the automatic imitation task. Next, they then carried out the task. Finally, participants completed a debriefing questionnaire, where they were asked what they thought the experiment was investigating (no subjects reported anything related to a direct relationship between subjective power and automatic imitation).

#### Participants

146 undergraduate students from the University of California, San Diego participated for course credit. All participants were right-handed and gave their written informed consent to participate. The University of California, San Diego Human Research Protection Program approved the study.

### Results

#### AI

Incorrect responses (5.91%) were excluded, as were all RTs smaller than 200 ms or greater 1000 ms (1.87%). Participants with error rates greater than 40% on the automatic imitation task were excluded from the main analysis (three participants). Among valid subjects, the number of correct responses was high (M = 95.10%; SD = 3.62%), and average RTs were fast (M = 450.92 ms, SD = 132.64 ms). RTs were analysed using a mixed repeated measures ANOVA, according to a 3 (Power: HP, LP, control) x 2 (Congruency: congruent, incongruent) x 2 (Trial Type: movement, effector priming) structure.

All main effects were significant. First, a main effect of Power, *F*(2, 140) = 3.45, *p* < .05, η^2^ = .045, showed that HP participants were slower (*M* = 477.03 ms; *SD* = 139.20 ms) than both LP participants (*M* = 446.12 ms; *SD* = 123.23 ms), *t*(112) = 1.84, *p* = .07, *r* = .117, and control participants (*M* = 424.43 ms; *SD* = 128.13 ms), *t*(82) = 2.34, *p* = .02, *r* = .193, but there was no difference between LP and control RTs, *t*(86) = 1.05, *ns*, *r* = .086. We also observed a main effect of Congruency, *F*(1, 140) = 396.81, *p* < .001, η^2^ = .077, which demonstrated that all participants were faster to respond to congruent trials (*M* = 427.96 ms; *SD* = 120.46 ms) than incongruent trials (*M* = 480.31 ms; *SD* = 138.28 ms). Finally, there was a main effect of Trial Type, *F*(1, 140) = 27.60, *p* < .001, η^2^ = .003, which revealed that all participants responded quicker to movement trials (*M* = 449.30 ms; *SD* = 131.82 ms) compared to effector priming trials (M = 457.59 ms; *SD* = 132.18 ms).

**Fig 9:**
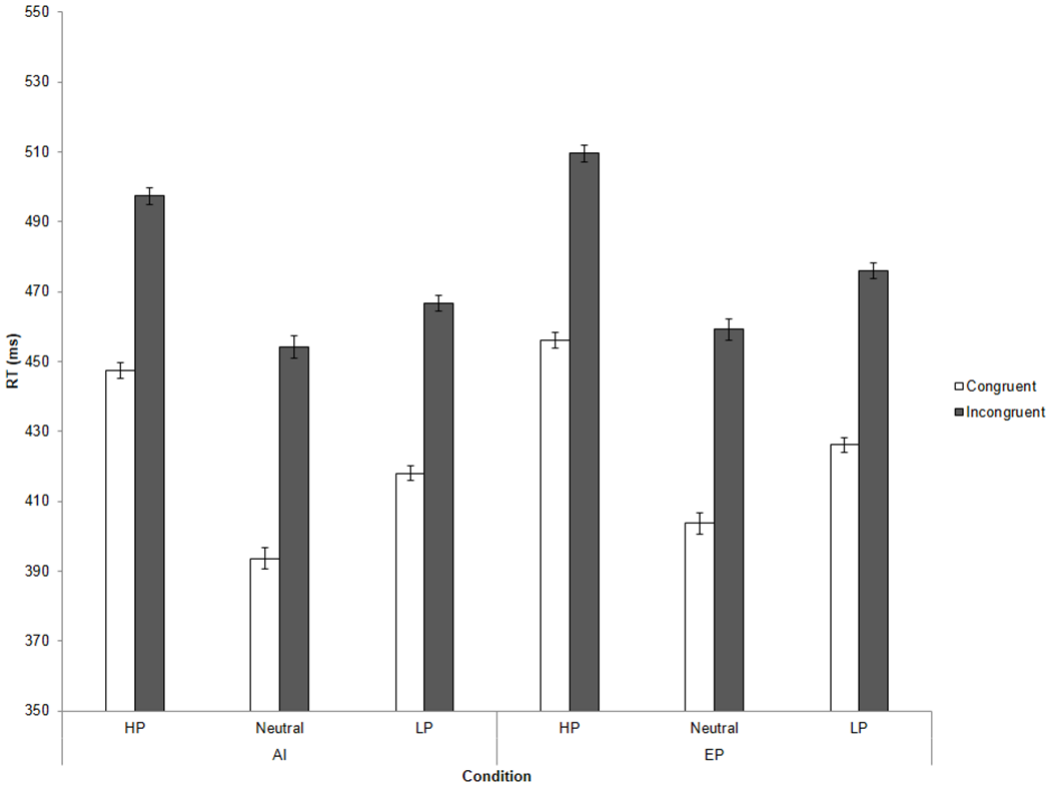
Reaction times (RTs) across all conditions of the finger-lifting task in Experiment 5. AI = automatic imitation, EP = effector priming. Error bars represent standard error of the mean (SEM).

However, we did *not* observe any interactions for Power x Congruency, *F*(2, 140) = .77, *ns*, η^2^ < .001, Power x Trial Type, *F*(2, 140) = 0.24, *ns*, η^2^ < .001, Congruency x Trial Type, *F*(1, 140) = .10, *ns*, η^2^ < .001, or Power x Congruency x Trial Type, *F*(2, 140) = .66, *ns*, η^2^ < .001 (see Fig 9).

### Discussion

Experiment 5 used a between-subjects design to investigate whether priming participants with a sense of high- or low-power would modulate their broad tendency towards automatic imitation. As in the previous studies, we found a congruency main effect but no interaction between congruency and power, suggesting that generalised feelings of power do not modulate the amount of automatic imitation. Unlike our previous experiments, there was a main effect of power on RTs, where high-power participants were significantly slower to react to the cue than those in the low-power condition. This effect is possibly due to the high-power prime leading to differences in attention (i.e., high-power may cause participants to pay less attention to the task trials) or motivation (i.e., high-power subjects may be less motivated to perform well).

Note however, similar to the previous experiments, no significant interactive effects were found for power and AI, suggesting that generalised feelings of high- or low-power do not strongly affect participant’s tendency to automatically imitate others.

## A Cross-Study Bayesian Analysis

The present paper reports five experiments with null results—an interaction between congruency and status/power was not found in any study. A key weakness of traditional statistical tests is that they only allow for the rejection of the null hypothesis but cannot indicate when the null hypothesis should be accepted as true [74]. In recent years, this weakness has led to an interest in the use of Bayesian hypothesis testing as an alternative method to assess the validity of the null hypothesis [75,76]. Bayesian hypothesis testing involves the calculation of Bayes Factors (BFs), which indicate the relative strength of evidence for one theory over another [77]. BFs can either indicate the odds of favouring the null hypothesis over the hypothesised alternative (BF_01_) or else indicate the favour of the experimental hypothesis (BF_10_). In our case, we sought to find the BF_01_, in order to assess whether our findings gave good evidence to accept the null hypothesis (and consequently, accept that there is no effect of social status/power on AI). BFs can range from zero to an infinite value, whereby a value of 1 does not favour either theory, and values above 1 indicate increasing evidence for one alternative over the other [77]. Jeffreys [78] suggests that odds greater than 3 should be considered as some evidence in favour of one hypothesis over another, whereas odds greater than 10 should be regarded as strong evidence.

In order to carry out Bayesian *t*-tests on our data, a congruency effect was calculated for each participant as the mean RT for incongruent trials minus the mean RT for congruent trials. A *t*-test was then carried out to compare congruency effects in the high-status/power and low-status/power conditions. The resulting *t* values were then entered into an online programme that calculated the BF_01_ for each experiment [76]. As recommended by the authors, these analyses are based upon the JZS Bayes Factor with the *r* scale set to 1. Table 6 shows the results of the analyses.

**Table 6:** Results of Bayesian t-tests for each of the 5 experiments.

Experiment	Mean Difference (CE)			Bayes Factor_(01)_
	Effect Size *d*	*t*-value	*p*-value	
1 (paired)	0.144	0.545	0.895	5.936
2 (paired)	0.046	-0.195	0.847	6.139
3 (paired)	0	0	1	3.817
4 (paired)	0.046	0.297	0.768	6.888
5 (independent, movement only)	0.041	-0.324	0.747	6.572

As can be seen in all 5 studies, the BF_01_ was greater than 3, giving us good evidence to favour the null hypothesis that social status/power does not modulate AI.

## General Discussion

The five experiments reported in this paper used a series of different manipulations to investigate whether social status (Experiments 1–3) or power (Experiments 4 and 5) had a moderating effect on automatic imitation (AI), as measured in a finger-movement task. No significant interactions were found between congruency and social status/power. Additionally, Bayesian hypothesis testing indicated that the null hypothesis should be favoured over the experimental hypothesis in all five studies. We now consider the possible reasons for the lack of a modulating effect of status or power on AI, as well as the other implications of our findings.

### Possible limitations in the status and power manipulations

One possible explanation for the lack of AI modulation by social status/power is to simply assume that our manipulations of the social status for other characters (Experiments 1–3) or of the amount of power experienced by our participants (Experiments 4 and 5) were unsuccessful. This seems unlikely, given that we used well-established and widely-used power manipulations (e.g., Galinsky, Gruenfeld, & Magee, 2003), and post-experiment questionnaires administered showed that participants associated the high-status/power individual with higher status/power characteristics.

Self-report measures, such as the manipulation check questionnaires used in Experiments 1–4, have a number of limitations—including being open to demand characteristics and having questionable validity in predicting behaviour [79–81]. However, note that Experiment 5 did not involve a questionnaire-based power manipulation check, and we still found a clear effect of power priming on participants’ performance on the finger-tapping task. Participants who had been primed with high-power subsequently showed slower RTs in both congruent and incongruent trials, compared to those who received low-power or neutral primes. This effect could likely be due to the fact that those in the high-power condition were less motivated to fully engage in the task, as they already felt a sense of achievement due to the prime. The data is in line with work by Hogeveen et al. [39], demonstrating that high-power primes reduced motor facilitation during action observation (which relates to slower motor responses). The fact that this effect was only found in Experiment 5 may be due to the fact that this was the only experiment that employed a between-subjects design, in which participants experienced only a single power-condition throughout the entirety of the study. Overall, the finding of power impacting overall motor behaviour (but not AI specifically) adds support to the claim that, at least with Experiment 5, the power manipulation was successful but had no effect on participants’ propensity to imitate.

Finally, the present series of studies represents a wide-ranging, cross-lab attempt to find an effect of power/status on AI, using a variety of established hierarchy manipulations. These ranged from learning about the status of the observed characters (Experiments 1 and 2), to behavioural demonstrations of the other characters superiority/inferiority at performing a task compared to the participant (Experiment 3), manipulating the amount of power participants had relative to other characters in a choice task (Experiment 4), and priming participants with a general sense of power (Experiment 5). While a failure to find an effect of power/status on any of these individual experiments could be due to the failure of one particular manipulation, it seems highly unlikely that none of these different manipulations succeeded in changing participants’ perceptions of power/status at all.

### Possible limitations in the automatic imitation task

A different category of possible explanations for our results relates to our preferred AI paradigm for these studies (over other BM approaches). The finger-tapping task is highly controlled and participants perform repeated trials as fast as possible. Thus, it is quite different from the kind of BM that individuals engage during real-life social interactions. Previous studies that have found an effect of status on mimicry [42,43] employed a naturalistic paradigm, where experimenters observed how much participants copied the movements of a confederate during a social interaction. While BM and AI are usually assumed to reflect the same underlying process, there is currently no conclusive evidence that these two processes rely on the same cognitive or neural mechanisms [10]. Therefore, effects found in one paradigm may not necessarily transfer to the other (for a similar argument, see [8]).

Another issue is that the finger movements are only one of a number of possible motor actions that have been used within the AI literature. Other studies have shown social modulations of AI using a task that requires movements of the whole hand (i.e., hand-opening or closing, e.g. [6,82]), which requires participants to pay greater attention to the observed hand action by making the start of that action the cue for participants to move. It is possible that more naturalistic motor responses (and greater emphasis on the observation of others’ actions) produce increased sensitivity to social modulations, compared to the finger tasks used in the current experiments. Also, an informed reader might note that the finger-tapping task used in Experiments 1–4 did not control for any effects of spatial compatibility, even though this was controlled for in Experiment 5 where we found similar null effects (using the vertically-oriented hand stimulus; see Fig 8).

Despite these potential limitations, the finger-movement tasks used here have previously been shown to be sensitive to other social modulators of mimicry. Several studies have used finger movements to demonstrate that AI increases when participants believe that the observed action is being generated by a human, as opposed to a robotic or virtual agent [83,84] or when the action they observe is biologically possible rather than biologically impossible [85]. These findings suggest that participants’ beliefs about the social nature of the observed stimuli can modulate the tendency to imitate. Further compelling evidence comes from studies showing that self-focus [86], prosocial and antisocial priming [4,56,73,87], self-construal [4], the facial expression of the observed character [49], and whether the observed character is a member of a racial in-group or out-group [49] can all effectively modulate AI in simple finger-movement tasks—even in cases where spatial compatibility effects are not controlled [4,49,56]. Furthermore, with our Experiment 5, we did control for spatial compatibility, and we still detected null results of a similar magnitude to Experiments 1–4, suggesting that the lack of control for spatial compatibility in those experiments cannot explain the failure to find an effect of status/power. Given these considerations, it seems unlikely that our failure to find a modulating effect of status or power on AI is due purely to the specific type of SRC task measure.

### The potential role of individual differences

Another factor that could impact AI modulation is individual differences in social characteristics. Both Cheng and Chartrand [42] and Ashton-James and Levordashka [43] found effects of status on mimicry only when taking into account individual differences. In the case of Cheng and Chartrand, they found that only those participants who were high in self-monitoring showed greater mimicry of the high-status actor than the low-status actor. In a similar way, Ashton-James and Levordashka found that participants who were high in trait narcissism mimicked the high-status actor significantly more than participants who were not high in trait narcissism. Self-monitoring and narcissism are both traits centred on the need for social acceptance, so it is not surprising that they are highly correlated [88]. And notably, two recent studies [51,89] have found that people with narcissistic traits show less AI in a finger-tapping task. While this might seem contradictory, these findings are compatible if we consider that people high in narcissism are also better at controlling their imitative behaviour, both in response to status cues (in the naturalistic context) and in response to incongruent stimuli (in the finger-tapping task).

In the current set of studies, only Experiment 4 measured individual differences by having participants complete a self-monitoring questionnaire [90]. We found that adding self-monitoring as a covariate had no effect on the relationship between power and mimicry (with no other effects). Thus, we did not replicate the findings described above. However, note that both our Experiment 4 (*n* = 31) and the previous studies (*n* = 27 in Obhi et al., [89]; *n* = 18 in Hogeveen and Obhi [51]; approximately *n* = 12 per group in Ashton-James and Levordashka [43]; approximately *n* = 10 per group in Cheng and Chartrand [42]) all have fairly low power to detect between-group differences or correlations between behaviour and personality traits [91]. A full assessment of the relationship between individual differences in self-presentation and the tendency to imitate will require further investigation.

### Accepting the null

Given the above-reviewed evidence, we argue that our power and status manipulations were robust and wide-ranging. Our AI task is also well established and has shown modulatory effects in previous studies. A series of five experiments with strong designs all demonstrated null results, and a cross-study Bayesian analysis clearly showed that these data argue in favour of accepting the null hypothesis. Thus, we conclude that manipulating social power and status does not modulate AI of finger movements in the context we describe. We now consider the implications of this conclusion for general theories of imitation and social interaction.

First, our results do *not* imply that power and status cues do not modulate *any* automatic social behaviour. Previous research has demonstrated that that social status can affect gaze following [36,92] and that being primed with a sense of power leads to slower motor responding ([39] and experiment 5 of the current paper). In addition, several other studies have shown more subtle effects of power and status on gaze fixation [93], emotion recognition [94] and autonomic nervous system responding [95]. These collective findings suggest that the null effects of power and status on AI found in our experiments are specific to mimicry (of finger movements), rather than generalising to other forms of social behaviour.

With this in mind, we suggest that power and status might have a greater impact when the movements involved are social gestures, like hand shaking [96,97], pointing [98] or other affective gestures (e.g., thumbs-up or down; [99,100]). It is also possible that contrasting high- and low-power related gestures, such as clenched versus unclenched fists [101], approach versus avoidance related movements [102–104] or dominant versus submissive postures [45,46,105] may have led to a greater effect of our power and status manipulations on AI. Such stimuli, which are more socially or emotionally “rich” than those used in the finger-movement task, might lead to automatic motor responding that dynamically integrates the context of the perceiver state, displayer state, and action itself [e.g., as with affective facial expressions; 47].

Another important factor to keep in mind is that mimicking those with a different status level as oneself is not necessarily the best way to increase affiliation and rapport. To use an obvious example, two people both adopting dominant postures are more likely to be perceived as in conflict. Tiedens and Fragale [46] found that participants more readily assumed a complementary posture to a confederate and preferred those complementary confederates (over those who mimicked them). This suggests that, in some cases, complementarity (rather than mimicry) is the more natural behaviour when interacting with those of a different social status.

It may be that complementary actions are more sensitive to manipulations of status and power than imitative actions. If so, this would explain the findings of the current experiments, as well as suggesting some intriguing directions for future research. As an example, to the extent that power-related movements and actions are over-learned, it is possible that one might be able to find a reverse stimulus compatibility effect of a similar type to that found for hand-shaking [96,97], in which dominant actions prime a submissive response (and vice versa). Manipulating the status or power of the participant compared to the observed character in such a situation might then lead to significant modulations of these effects.

It is also important to consider the theoretical implications of these results. The STORM model [3], along with other mimicry frameworks [2,8] have suggested that people should show greater AI of high-status individuals compared to low-status individuals, if greater mimicry leads to greater affiliation. However, this argument depends on three key assumptions: First, mimicry will lead to increased liking in all situations. Second, people always desire to be liked by those with high-status. And third, AI tasks such as the finger tapping and lifting (as used here) are susceptible to strategic modulation, based on the identity of the person being imitated. Therefore, the null findings from the current experiments could be explained by a breakdown in one or more of these assumptions.

On the first assumption, it may be that mimicry of intransitive movements does not always lead to increased affiliation. Alternatively, as discussed above, it is possible that in the case of status and power, complementary gestures lead to a greater sense of affiliation than mimicked gestures. On the second assumption, work by Fiske and others [106–108] has shown that people often rate those with higher status as competent but unlikeable, particularly when they are in competition with them (as in Experiments 3 and 4 of the current paper). And on the third assumption, although previous studies have shown that social factors can impact AI, none of these factors required participants to distinguish between two characters on the basis of such an abstract level as status and power. Therefore, it might be possible that the cognitive mechanisms underpinning AI are not sensitive to the strategic planning necessary for status and power (while still being sensitive to other less cognitively demanding, yet still social factors). Further research will be needed to distinguish between these possibilities, but at present, it is possible that mimicry is used in a “Machiavellian” manner—albeit in a more nuanced manner than previously thought.

Overall, our studies suggest that theories on the social modulation of mimicry must take into account how the interaction partner is likely to interpret the behaviour of the mimicker. For instance, a recent study by Kavanagh, Suhler, Churchland, and Winkielman [48] demonstrated that mimicking an unpleasant person can cause third-party observers to rate the mimicker as being less socially competent (compared to when they do *not* mimic the observer). Such findings, along with the experiments outlined in this paper, demonstrate the need for theoretical mimicry models to take into account the complex network of hierarchies, goals, and expectations that surround dyadic interactions.

### Conclusions

In conclusion, the current set of studies examined the extent to which status and power modulate AI. Despite using a wide range of well-established manipulations and paradigms, we found no evidence for a significant effect on AI of intransitive finger movements. Moreover, Bayesian hypothesis testing indicated that all experiments favoured the null hypothesis.

Generally, these findings suggest that mimicry is not always engaged in cases where the observed behaviour is non-goal-directed. However, they leave open the possibility that *other* automatic behaviours may be utilized in cases, where social cues may be more complex.
